# Spatial Platform for Periodontal Ligament Angulation and Regeneration: In Vivo Pilot Study

**DOI:** 10.3390/jfb16030099

**Published:** 2025-03-13

**Authors:** Min Guk Kim, Do-Yeon Kim, Hyoung-Gon Ko, Jin-Seok Byun, Joong-Hyun Kim, Chan Ho Park

**Affiliations:** 1 Department of Dental Biomaterials, School of Dentistry, Kyungpook National University, Daegu 41940, Republic of Korea; 2 Advanced Dental Device Development Institute (A3DI), Kyungpook National University, Daegu 41940, Republic of Korea; 3 Department of Dental Science, Graduate School, Kyungpook National University, Daegu 41940, Republic of Korea; 4 Department of Pharmacology, School of Dentistry, Kyungpook National University, Daegu 41940, Republic of Korea; 5 Department of Oral Anatomy and Developmental Biology, College of Dentistry, Kyung Hee University, Seoul 02447, Republic of Korea; 6 Department of Oral Medicine, School of Dentistry, Kyungpook National University, Daegu 41940, Republic of Korea; 7 Non-Clinical Evaluation Center, Osong Medical Innovation Foundation (KBIO Health®), Cheongju 28160, Republic of Korea

**Keywords:** periodontal ligament (PDL), additive manufacturing, periodontal tissues, tissue engineering, regenerative medicine

## Abstract

The periodontal ligament (PDL) is a fibrous connective tissue that anchors the tooth-root surface to the alveolar bone with specific orientations. It plays a crucial role in functional restoration, optimal position stabilities, biomechanical stress transmission, and appropriate tissue remodeling in response to masticatory loading conditions. This pilot study explored spatial microarchitectures to promote PDL orientations while limiting mineralized tissue formation. A computer-designed perio-complex scaffold was developed with two parts: (1) PDL-guiding architectures with defined surface topography and (2) a bone region with open structures. After SEM analysis of micropatterned topographies on PDL-guiding architectures, perio-complex scaffolds were transplanted into two-wall periodontal defects in the canine mandible. Despite the limited bone formation at the 4-week timepoint, bone parameters in micro-CT quantifications showed statistically significant differences between the no-scaffold and perio-complex scaffold transplantation groups. Histological analyses demonstrated that the PDL-guiding architecture regulated fiber orientations and facilitated the functional restoration of PDL bundles in immunohistochemistry with periostin and decorin (DCN). The perio-complex scaffold exhibited predictable and controlled fibrous tissue alignment with specific angulations, ensuring spatial compartmentalization for PDL tissues and bone regenerations. These findings highlighted that the perio-complex scaffold could serve as an advanced therapeutic approach to contribute periodontal tissue regeneration and functional restoration in tooth-supporting structures.

## 1. Introduction

Periodontal ligament (PDL) tissues are fibrous connective tissues that are anchored to mineralized tissues with Sharpey’s fibers, and they display spatiotemporal peculiarities, with perpendicular or oblique orientations to tooth-root surfaces within 100–300 µm PDL interfaces [1,2]. The angular principal fiber bundles generate various functional responses to effectively ensure the positional stability of teeth, distribute various biomechanical stresses, and promote tissue remodeling by transmitting mechanical stimulation during mastication [3,4]. However, periodontitis, a highly prevalent inflammatory infectious disease initiated by the microflora (or its metabolic products), leads to the destruction of the periodontal tissue via the resorption of alveolar bone and the attachment loss of PDL bundles [5,6,7,8]. In more severe cases of periodontal disease progression, dental prostheses such as dentures or dental implants may be required to replace the masticatory function of natural teeth after tooth extraction [9,10]. Therefore, the regeneration of periodontia lost as a result of disease or traumatic injuries and their structural integration by mediating angulated PDL anchorage represent a key strategy to restore functions in order to support and preserve natural teeth [2,11,12].

Among the various tissue engineering accomplishments achieved using engineered three-dimensional (3D) platforms [13,14,15], additive manufacturing (AM) technology has been widely used to promote periodontal tissue neogenesis with regional compartmentalization of PDL and alveolar bone [16,17,18,19]. Our recent studies demonstrated that surface artifacts created during the AM procedure can guide the angulation of human PDL cells with a high cell population in vitro [20,21] and promote the formation of fibrous tissue bundles with specific alignments in a subcutaneous transplantation model using harvested rat PDL cells [22]. Based on the achievements made with optimally characterized surface topography, this pilot study developed PDL-guiding architectures within the perio-complex scaffolding system to facilitate the angular alignment of ligamentous tissues and restore tooth-supportive function in two-wall periodontal defects.

## 2. Materials and Methods

### 2.1. Customized Perio-Complex Scaffolds Using a Two-Wall Periodontal Defect Model

**Figure 1:**
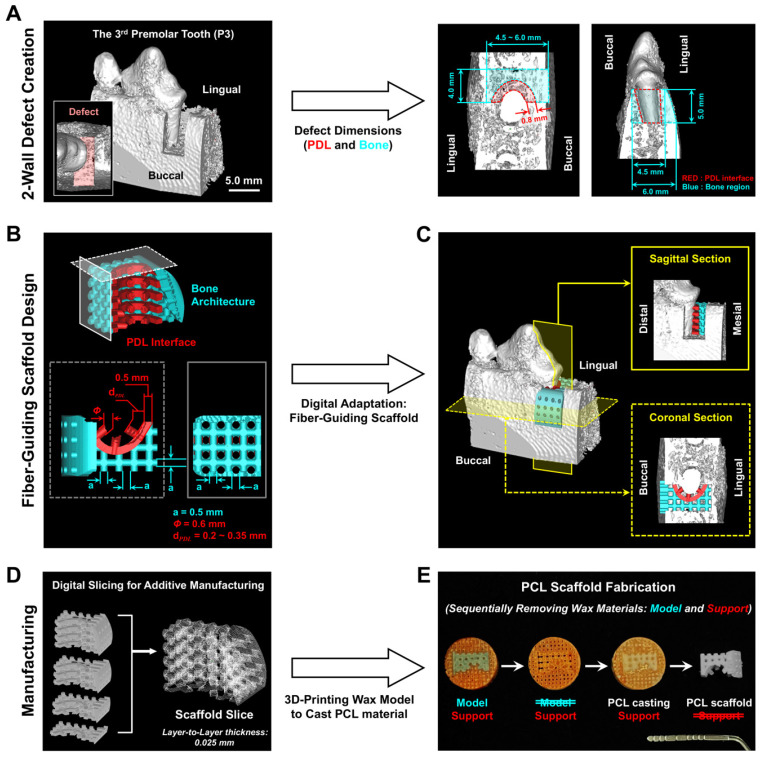
Computer-designed scaffold fabrication using the 2-wall periodontal defect in the canine model. (**A**) Micro-CT images were three-dimensionally reconstructed, showing the standardized periodontal defect with specific dimensions. (**B**) The customized perio-complex scaffolding system was designed with PDL-guiding architectures with a 0.2~0.35 mm interface and a 0.5 mm interconnective structure (red part) linked to the bone region, designed with 0.5 mm diameter struts and interconnective pores (blue part). (**C**) The schematic figure shows the digital adaptation using 3D defect model images and the designed perio-complex scaffold with a sagittal section and a coronal section, which can provide information on the contact with the tooth-root surface. (**D**) The 2D digital slicing procedure was performed with 25.40 μm intervals to generate a microgroove-patterned surface to control the angular orientations of fibrous connective tissues. (**E**) After additively manufacturing the wax mold and selectively dissolving the model wax material with ethanol, 25% poly-ε-caprolactone (PCL) in 1,4-dioxane was cast into the support wax mold, and PCL scaffolds could be obtained by removing the red wax material.

Using the micro-computed tomography (micro-CT) scanning dataset around the 3rd premolar tooth (P3) of a mandible in a canine cadaveric model, a 2-wall periodontal defect was digitally designed with 5.0 mm height, 4.5–6.0 mm depth (buccal-to-lingual distance), and 4.0 mm width (bony wall-to-tooth center) (Figure 1A). In this 2-wall defect, perio-complex scaffolds were designed with two specific compartments for bone regeneration in the bone architecture and fibrous tissue alignments in the PDL interface (Figure 1B,C) via Solidworks 2019 (Dassault Systèmes Solidworks Corporation, Waltham, MA, USA). Prior to manufacturing wax-based casting molds using the 3D printing system (Solidscape 3Z Pro; Solidscape^®^, Inc., Merrimack, NH, USA), the specific layer thickness during the digital slicing step was determined to be 25.40 μm (Figure 1D), representing the optimized microgroove pattern intervals to organize fibroblast-like cells and fibrous tissues with specific angulations [20,21]. Briefly, after the dual-wax printing system manufactured the mold with both modeling and supporting materials, the modeling material (blue wax) was dissolved with ethanol to obtain the final mold based on a supporting red wax material (Figure 1E). Then, 25% poly-ε-caprolactone (PCL; MW 43~50 kDa, Polysciences Inc., Warrington, PA, USA) in 1,4-dioxane was cast into the sacrificial red mold, the mold was freeze-dried for 7 days, and cyclohexane (Sigma-Aldrich, St. Louis, MO, USA) was utilized to remove the sacrificial red wax at 35–37 °C for 2 days to obtain the PCL scaffold (Figure 1E).

### 2.2. Surface Characterization of PDL-Guiding Architectures in a Perio-Complex Scaffold

**Figure 2:**
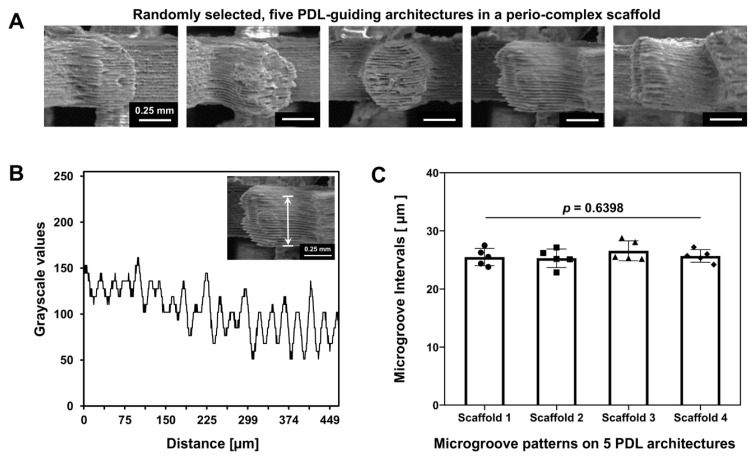
Qualitative and quantitative analyses using SEM images. (**A**) SEM images providing the micropatterned topographies on PDL-guiding architectures. (**B**) Microgroove intervals profiled by crossing the surface of PDL-guiding architectures for quantitative assessments. (**C**) Based on data for the pattern profiles, five PDL-guiding architectures per scaffold were randomly selected and the microgroove intervals were measured, and four different scaffolds were compared to validate the statistical similarity and reproducibility of these microgroove patterns. For the statistical analysis, one-way analysis of variance (ANOVA) with Bonferroni post hoc tests were performed, and statistical significance was set at *p* < 0.05 (n_PDL_ = 5/scaffold; *p* = 0.6398).

The topographical microstructures in the perio-complex scaffold were characterized using a field emission scanning electron microscope (FE-SEM; SU8010, Hitachi, Tokyo, Japan) at 20 kV and analyzed using ImageJ 1.54g software (National Institutes of Health, Bethesda, MD, USA) (Figure 2A). Based on the topographical profiles on a single PDL-guiding architecture, microgroove pattern intervals were quantitatively assessed (Figure 2B) and statistically calculated with four different PDL-guiding architectures from an individual scaffold (Figure 2C).

### 2.3. Surgical Creation of 2-Wall Periodontal Defects and Scaffold Transplantation

The canine mandibular defect model was established using two healthy beagle dogs (1.5 years of age with a weight of approximately 14 kg) from Woojung Bio (Hwaseong-si, Gyeonggi-do, Republic of Korea). Individual animals were housed at the standard temperature (21–25 °C) with free access to water and were fed 300 g of food each day. All procedures for this animal study followed the approved protocol by the Institutional Animal Care and Use Committee, KBIO Health (IACUC #: KBIO-IACUC-2022-157).

**Figure 3:**
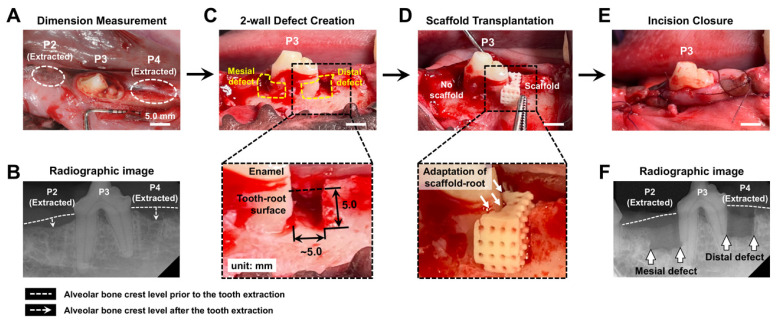
Surgical creation of the 2-wall periodontal defect and scaffold transplantation after tooth extraction socket healing. (**A**,**B**) After extracting the 2nd and 4th premolar teeth (P2 and P4), the X-ray radiograph showed natural bone healing within 12 weeks. (**C**) Periodontal 2-wall defects were surgically created around the mesial and distal roots of the 3rd premolar tooth (P3) with specific dimensions, and curettes were utilized to remove the cementum and periodontal ligaments on the tooth-root surface. (**D**) One defect received the perio-complex scaffold transplantation with the geometric adaptation between the scaffold and tooth-root, while the other was left vacant. (**E**,**F**) After the flap was sutured in position, X-ray radiography provided the experimental defect sites around the P3 tooth. White arrows indicate horizontal defect margins, and white dashed lines represent the borderline of the original bone crest level. Scale bar, 5.0 mm.

For pre-anesthesia induction, zoletil (5 mg/kg) and xylazine (2 mg/kg) were intramuscularly (IM) injected, and inhalation anesthesia was conducted to maintain anesthesia with isoflurane (<3%) during two individual surgeries: tooth extraction and the defect model’s creation. After extraction of the P2 and the P4 teeth (Figure 3A), alveolar bone was naturally regenerated to fill tooth extraction socket defects within 12 weeks (Figure 3B). To prevent scaffold breakage during the press-fit procedure, additional extra space was surgically created in the bony defects around the mesial and distal roots of the P3 tooth (mesiodistal width: approximately 5.0 mm; buccolingual width: 4.0 mm; depth: 5.0 mm; Figure 3C and Figure S1). Following optimal defect preparation, cementum and PDL were physically removed from the tooth-root surface by means of a Gracey curette. During the scaffold transplantation, PDL-guiding architectures were preserved as computer-designed structures, and the adaptability of the scaffold-tooth root surface was ensured by filling the marginal space between the scaffold and bone defect surface with pulverized bone architectures of scaffolds. After gingival closure, butorphanol (0.4 mg/kg; Myungmoon Pharm Co., Ltd., Seoul, Republic of Korea) was injected subcutaneously to provide pain relief.

The post-operative management included the administration of antibiotics (cefazoline sodium, 20 mg/kg), analgesics (Meloxicam, 0.04 mL/kg), a soft diet, and daily rinsing using a 2% chlorohexidine solution. In addition, transplantation sites were monitored daily for clinical signs of infection, edema, inflammation, and tightly maintained suture closure until surgical sutures were removed. For the euthanasia at 4 weeks, zoletil (5 mg/kg) and xylazine (2 mg/kg) were administered via intravenous (IV) injection into the cephalic vein. After euthanized animals received an IV injection with a large dose of potassium chloride (KCl, 40 mL), exsanguination was performed by severing the carotid artery.

### 2.4. Micro-Computed Tomgoraphy (Micro-CT) Assessments

After sacrificing the animals and fixing the harvested canine mandibles in 10% neutral buffered formalin for 48 h at room temperature, micro-computed tomography (micro-CT; Skyscan 1272, Bruker micro-CT, Kontich, Belgium) was used to scan all fixed specimens with an accelerating potential of 70 kV, a beam current of 142 µA, a voxel size of 13.18 µm^3^, and a 0.5 mm aluminum filter. Based on the Hounsfield Unit (HU) grayscale values, mineralized tissue formation was quantitatively analyzed using a CT Analyzer (Skyscan) and Data Viewer (SkyScan). Using the reference notches on the tooth-root surface, the volumes of interest (VOIs) were digitally created with the following specific dimensions in the defect site: height—approximately 5.0 mm; mesiodistal length—5.0~6.0 mm; buccolingual length—5.0~6.0 mm (Figure S2). After the image reconstruction process using NRecon (Skyscan) with the Hounsfield Unit (HU)-based grayscale values, visualization and quantification assessments were performed using the CT Analyzer and Data Viewer (SkyScan).

### 2.5. Histological and Immunohistochemical Analyses for Oriented Ligament Formations

After scanning with micro-CT, specimens were decalcified using a RapidCal™ Immuno solution (BBC Biochemical, Mount Vernon, WA, USA) for 4 weeks to ensure the complete removal of mineralized components. The tissues were paraffin-embedded and sectioned within approximately 5 µm thick slices using a microtome for subsequent staining and microscopic evaluation. The hematoxylin and eosin (H&E; BBC Biochemical) staining method was performed for biological and pathological assessments of regenerated fibrous connective tissues. Moreover, collagen bundle formation and the fiber orientations around teeth were qualitatively visualized with Picro-Sirius Red (Abcam, Inc., Cambridge, MA, USA) staining.

For analyses of functional restoration, immunohistochemistry with periostin and decorin (DCN) antibodies (1:200 dilution; Abcam, Inc.) was performed using a rabbit-specific horseradish peroxidase/diaminobenzidine (HRP/DAB) detection IHC kit (Abcam, Inc.). The stained sections were examined microscopically to assess the levels and distribution of periostin and DCN in the regenerating tissue.

### 2.6. Statistical Analysis

IBM SPSS Statistics 27.0 (IBM SPSS Statistics, Chicago, IL, USA) was utilized for statistical analyses with means ± standard deviations. For multiple comparisons of microgroove patterns with statistics, one-way analysis of variance (ANOVA) with Bonferroni post hoc tests was performed for multiple group comparisons (Figure 2C). For the quantitative assessments of mineralized tissue formation in micro-CT, the Student *t*-test was utilized (n = 4/group). Statistical difference was significant if the *p*-value was less than 0.05 (α = 0.05).

## 3. Results

### 3.1. Customized Perio-Complex Scaffold Design Based on Two-Wall Periodontal Defects

The layer-by-layer AM technique created microgroove patterns on the PDL-guiding architectures at 25.40 μm intervals, which were computationally designed for fibrous cell and tissue alignments [20,22]. Although the linear measurement using SEM images was limited to a consistent interval (25.40 μm) due to the cylindrical structure of the PDL-guiding architectures (Figure 2A), five different PDL-guiding architectures (n_PDL_ = 5/scaffold) were randomly selected, and the pattern intervals were measured (Figure 2). Four different scaffolds were compared to validate the reproducibility of microgroove patterns, demonstrating no statistically significant differences (*p* = 0.6398; Figure 2B,C), indicating that AM can provide highly reproducible and controllable surface structures during manufacturing (Figure 2C).

### 3.2. Volumetric Analyses of Bone Formation in Micro-CT

**Figure 4:**
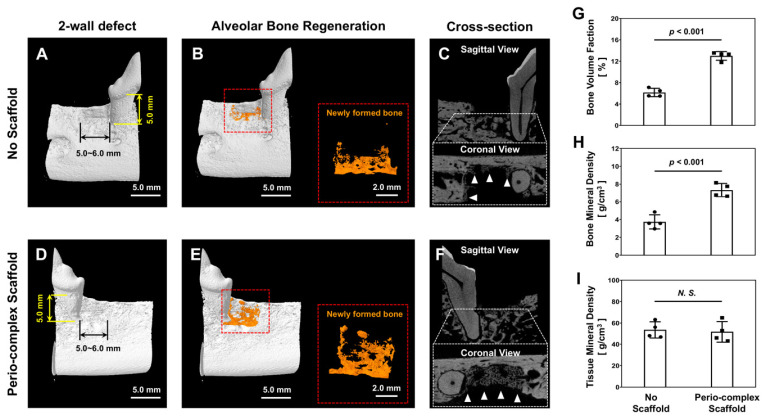
The qualification and quantification assessments of micro-CT to evaluate mineralized tissue formation into 2-wall periodontal defects. (**A**,**B**,**D**,**E**) Three-dimensional reconstructed micro-CT images representing mineralized tissue formation (orange color) in the defect sites. (**C**) In cross-sectional images, both the sagittal and coronal views show that there was minimal bone formation around the tooth structure, but (**F**) the perio-complex scaffold group had more qualitative and quantitative bone regeneration in the defect site. (**G**,**H**,**I**) Using bone parameters, namely bone volume fraction, bone mineral density, and tissue mineral density at the defect sites, mineralized tissues were statistically analyzed and assessed quantitatively at 4 weeks. (**G**,**H**) The perio-complex scaffold group showed statistically significant differences compared with the no-scaffold group regarding bone parameters. (**I**) However, the tissue mineral density displayed by the two groups was not statistically significantly different, indicating that the quality of regenerated bone tissue was similar in both groups. For the statistical analysis, a two-tailed independent-sample *t*-test was performed, and statistical significance was set at *p* < 0.05 (n = 4/group). White triangles indicate regenerated bone tissues in both groups.

Four weeks after sacrificing the animals and harvesting specimens, micro-CT images were analyzed regarding mineralized tissue formation in the defect sites. Although the 4-week timepoint represented a relatively short timeframe for the created alveolar bone defects in the canine model to completely heal, scaffold transplantation defect sites showed statistically significant differences regarding bone parameters (Figure 4). Compared with the no-scaffold group (n_vacancy_ = 4), the perio-complex scaffold group (n_scaffold_ = 4) displayed spatiotemporal bone tissue formation (Figure 4A–F). In quantification assessments, the perio-complex scaffold group showed statistically significant differences in the bone volume fraction (BVF) and bone mineral density (BMD), indicating regenerated bone volume (BV) and contents (BCs) in the defect sites (regions of interest: ROIs), respectively (Figure 4G,H). Regarding the quality of this tissue, tissue mineral density (TMD) was calculated according to regenerated BC and its volume and was statistically similar in both groups (Figure 4I). Although this pilot study was able to calculate the statistical difference between both groups, the short timeframe was insufficient for the perio-complex scaffold group to display promoted bone regeneration in the defects. Furthermore, the absence of biological factors could hinder the rapid quantitative formation of high-quality bone tissue, which is required to support teeth as well as newly formed PDL structures.

### 3.3. Histological and Immunohistochemical Analyses

**Figure 5:**
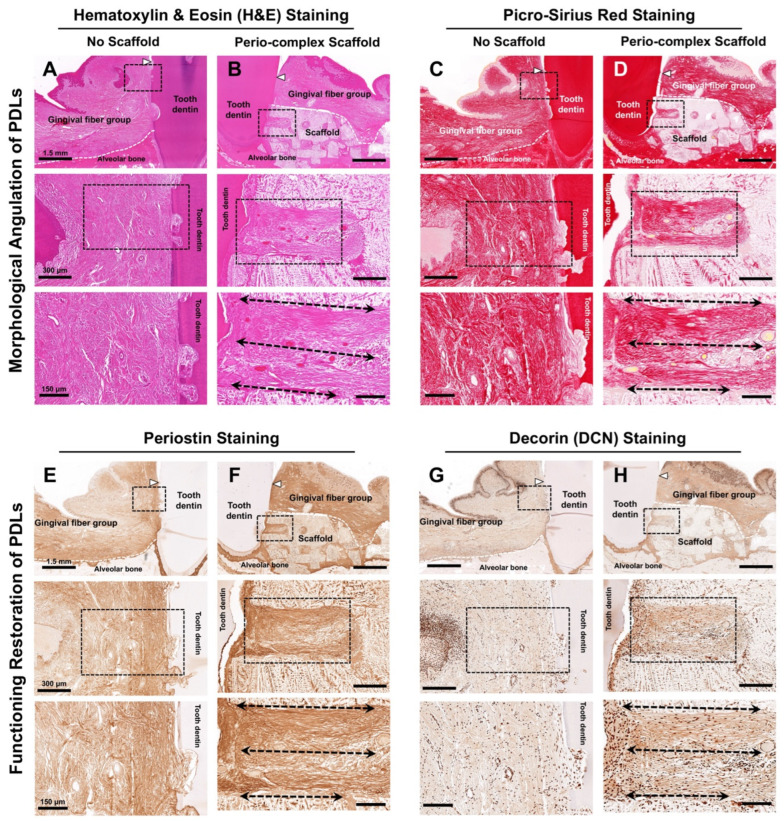
Histological analyses of PDL orientations and immunohistochemistry analyses to determine the regeneration of functioning periodontal ligament (PDL) tissues in the no-scaffold and perio-complex scaffold groups. (**A**,**B**) Perio-complex scaffolding system facilitated the control of fibrous connective tissue orientation, with perpendicular angulation to the tooth-root surface, while the no-scaffold group showed random organization of fibrous tissues around the tooth through H&E staining. (**C**,**D**) Collagen formation was qualitatively analyzed with regard to the angular orientation in both groups via Picro-Sirius Red staining. (**D**) In the perio-complex scaffold group, collagen bundles were formed perpendicular to the tooth-root surface, following the direction of the microgroove pattern created on the PDL-guiding architectures, whereas (**C**) the no-scaffold group displayed a random orientation or parallel alignment of the collagen structures to the tooth-root surface. (**E**,**F**) The expression of periostin staining represented the functional restoration of regenerated PDL tissues around the tooth root in the ligamentous region. (**F**) The expression level in the perio-complex scaffold group was significantly higher with fibrous angulations in the scaffold than in (**E**) the no-scaffold group. (**G**,**H**) The DCN antibody molecule was utilized to determine the quality and organization of collagen fibers, which are critical for the regeneration and functional restoration of PDL tissues. (**G**) The no-scaffold transplantation group showed significantly lower expression levels of DCN around the tooth structures, while (**H**) the perio-complex scaffold group displayed higher expression with aligned collagenous tissue bundles on the PDL-guiding architectures. White triangles indicate the cementoenamel junction (CEJ), white dashed lines represent the borderline of the gingival tissues, and black dashed lined arrows indicate the orientation of the fibrous tissue. Scale bars: 1.5 mm for top panels, 300 μm for middle panels, and 150 μm for bottom panels.

Using histology or histomorphometry, fibrous connective tissues linked to the tooth-root surface were angularly analyzed on a spatial platform in the fiber-guiding scaffold. The eosin stain was used to non-specifically stain proteins in the cytoplasm, along the cell membrane borders, in red blood cells, and in extracellular structures (Figure 5A,B), while Picro-Sirius Red staining was utilized to determine collagen formation or morphologically visualize collagen positivity (Figure 5C,D) in both groups. In the no-scaffold group, there was a critical limitation in maintaining the space for alveolar bone formation around tooth structures and the epithelial downgrowth of fibrous tissues, with this being randomly organized (Figure 5A,C). In contrast, in the perio-complex scaffold group, collagenous fiber bundles were angularly organized in a perio-complex scaffold, which had fiber-guiding architectures with microgroove patterns perpendicular to the tooth-root surface (Figure 5B,D). According to our results, the created topographical features on the surface of the fiber-guiding architectures could promote fibrous tissue formation in a scaffold, as well as regulating the angular alignments with structural similarity to the natural PDL tissues.

PDL maturity, integrity, and functionality at 4 weeks after treatment were indirectly determined and evaluated using immunohistochemical biomarkers—periostin and DCN molecules around the periodontal defect regions (Figure 5E–H). Interestingly, aligned PDL fibrous tissues in the perio-complex scaffold group showed high expression levels and greater immunoactivities of these two biomarkers (Figure 5F,H) compared with the fibrous tissue formation with a random organization and epithelial downgrowth in the no-scaffold group (Figure 5E,G). In other words, the immunohistochemical analyses demonstrate that engineered ligamentous bundles using the perio-complex scaffolds with topographic architectures could consequently promote biomechanical–functional restoration within the specific microenvironments within a short timeframe (Figure 5E–H).

## 4. Discussion

PDL tissues have crucial functions in optimizing the positional stability of teeth, transmitting and absorbing various stresses under masticatory/occlusal loading conditions, and promoting tissue remodeling via mechanical stimulation [23,24]. For the functional implementation of periodontal complexes, PDL tissues essentially require spatiotemporal orientation, which can be regionally classified for biomechanical senses and optimal responses [25]. Although various engineering approaches have been rapidly developed for periodontal tissue regeneration [26,27,28], it is still challenging to angularly organize regenerated PDL tissues within spatial compartmentalization in order to achieve functional restoration as a tooth-supportive structure.

In the AM process, the post-processing operation is generally the key step in removing artifacts to improve surface quality and mechanical properties [29], because the layer-by-layer manufacturing process inherently creates stair-step or topographic artifacts on the surface [30,31]. However, our previous studies reinterpreted these surface artifacts as tissue-guiding architectures with structural significance to control cell orientation in vitro and tissue alignments in vivo [20,21,22]. This pilot study used a canine model to validate the functional restoration of PDL tissues, which was critically identified as a limitation in preliminary research. These findings could be attributed to the PDL-guiding architectures, which could serve as a spatial platform to control tissue angulation for PDL functional restoration around tooth structures. After creating the topographical architectures depicted in Figure 1, quantitative and qualitative characterization of the microgroove patterns and their intervals was performed, which could be precisely defined at 25.40 μm during the digital slicing step (Figure 2). Prior to the scaffold transplantation, mock surgeries were performed to secure space for scaffold implantation with high adaptation ability, ensure intact positions in the designed defects, and prevent physical damage during transplantation, and the procedure resulted in the optimization of the final defect size with additional space (a margin of approximately 1.0 mm) mesially or distally around the P3 tooth (Figure S1).

The visualized analyses showed limited bone regeneration due to the relatively short timeframe and the absence of growth factors to enhance tissue regeneration, which inevitably led to slower growth rates of alveolar bone (Figure 4B,E). Nevertheless, the micro-CT quantification demonstrated that the two groups showed statistically significant differences with regard to BVF and BMD, which were calculated according to the total volumes of the defect site (TV; Figure 4G,H). The qualification of regenerated bone, which was calculated based on BV instead of TV, showed no statistically significant differences between the groups, as indicated by TMD (Figure 4I). Based on the results, this pilot study without bioactive molecules demonstrated that the perio-complex scaffold could typically contribute to volumetric bone formation quantitatively and qualitatively, with statistically significant differences. Using histology and immunohistochemistry, fibrous tissue formation, tissue alignments with angulations, and functional restoration of fibrous connective tissues were analyzed biologically and pathologically as tooth-supporting structures. While the no-scaffold group showed random organization of the fibrous tissue around the tooth (Figure 5A,C), the perio-complex scaffold group displayed fibrous connective tissues that had regenerated in the direction of the PDL-guiding architectures, designed and manufactured perpendicular to the tooth-root surface (Figure 5B,D). Although hematoxylin staining was entirely absent due to there being more eosinophilic samples caused by hydrochloric acid and formic acid in the rapid decalcification reagent [32,33], eosin staining was able to highlight tissues in order to allow morphological assessments using the H&E staining method (Figure 5A,B). For the specific visualization of newly formed collagen networks in the periodontal defects, Picro-Sirius Red staining could be an essential tool to provide quantifications of collagen matrix development and morphologically analyze the angular organization of regenerated PDL tissues (Figure 5C,D). Corresponding to the staining results, collagen networks were formed with critical directionality and allowed us to simply analyze Type I and III collagen matrix formation, which constituted fibrous connective tissues including PDL tissues (Figure 5).

Although tooth-supporting function may be lost in the defect sites by surgically creating periodontal two-wall defects around the P3 teeth, the periodontal tissues around the furcation involvement area and the lingual region could play a compensable role by generating responses for adaptation to mechanical and biological stimuli. Therefore, the biomechanical tests to simulate mastication or occlusion could be less effective and derive inaccurate results when evaluating the functional restoration of regenerated PDL tissues. Instead, immunohistochemistry was considered as an indirect assessment to investigate the functional restoration of regenerated PDL tissues using periostin and DCN antibodies (Figure 5E–H), which could typically express functioning or functionalized PDL tissues [34,35,36]. Periostin is a matricellular protein predominantly expressed in PDL tissues and essential for PDL homeostasis, tissue integrity, and the biomechanical functions that support tooth movement during mastication or occlusion [37]. In addition, DCN is a small leucine-rich proteoglycan that plays a pivotal role in collagen fibrillogenesis, tissue repair, and integrated structure maintenance of the extracellular matrix [36]. Both biomarkers are critically associated with fibrous connective tissue formation in periodontal complexes, the alignment of collagen bundles, and the functional restoration of regenerated PDL tissues around tooth structures [36,38,39].

This pilot study concentrated on the specific alignments and functional restoration of regenerated PDL tissues. Its limitations include the use of an insufficient timeframe to induce periodontal tissue regeneration and wound healing (4 weeks), as well as the limited number of subjects, making it difficult to standardize physiological variations among individuals. Nevertheless, our investigation demonstrated that the physically controlled orientation and spatial organization of PDL tissues by PDL-guiding architectures (the topographic strategy) could critically lead to high expression levels of periostin and DCN, which were significantly correlated with the adjacent natural PDL tissues in the apical and lingual regions of the tooth (Figure 5E–H). That is, while various studies on periodontal tissue regeneration have generally relied on responses to stimuli transmitted by mastication or occlusion to naturally align PDL tissues for their functions [23,40], the perio-complex scaffold with PDL-guiding architectures facilitated the angular organization of fibrous connective tissue bundles with specific directionality, maintained the orientation of PDL cells and tissues, and significantly contributed to the gradual functional restoration within the periodontal environment, as assessed via immunohistochemistry. Further studies are required to incorporate bioactive molecules such as growth factors with a perio-complex scaffolding system to accelerate the regeneration of cementum and alveolar bone to induce tissue integration for the purposes of preserving natural teeth.

Although this pilot study provides promising insights into periodontal complex regeneration, we believe that the typical limitations of this study should be acknowledged due to the relatively short experimental duration (4 weeks), such as insufficient volume of alveolar bone regeneration, the absence of morphological integration by the anchorage of Sharpey’s fibers, and direct biomechanical assessments to validate the biomechanical–functional adaptation of the regenerated periodontal complex. Therefore, to overcome these limitations and facilitate a paradigm shift in dental implantology, future studies are required to (1) enhance tissue regeneration using bioactive factor-mediated approaches, (2) extend the experimental duration to enable the maturation of periodontal complexes including long-term stability and tissue integration, and (3) evaluate biomechanical load distribution or response generation in regenerated periodontal structures for tooth-supportive functional restoration. Ultimately, these challenges would be crucial to establish clinically viable studies for periodontal tissue engineering strategies by prioritizing functional periodontal complex neogenesis over the conventional treatment using dental implants.

## 5. Conclusions

This pilot study concentrated on the specific alignments and functional restoration of regenerated PDL tissues, even though it could be a challenge to induce periodontal tissue regeneration and wound healing within a limited timeframe (4 weeks), while the limited number of subjects made it difficult to standardize physiological variations among individuals. The perio-complex scaffold with a PDL-guiding architectures facilitated the angular organization of fibrous connective tissue bundles with specific directionality and significantly contributed to the gradual functional restoration within the periodontal environment, as assessed by means of immunohistochemistry.

## Data Availability

The original contributions presented in this study are included in the article/supplementary material. Further inquiries can be directed to the corresponding authors.

## Supplementary Materials

The following supporting information can be downloaded at https://www.mdpi.com/article/10.3390/jfb16030099/s1, Figure S1: Optimization of defect dimension for scaffold accessibility. To avoid the destruction of perio-complex scaffolds during the transplantation, the preliminary mock surgery was executed. (a) The 3D-printed model was manufactured with the digital designed model (yellow area) and (b) extra-space was created to access a scaffold (red area). By practical testing, (c) it was determined that an extra-space of 2.0 mm (red) roughly facilitated scaffold transplantation without damaging the PDL-guiding architectures in a perio-complex scaffold. Figure S2: The creation of region-of-interest (ROI) in the 2-wall periodontal defect sites in no scaffold and perio-complex scaffold groups. From the tooth-root, ROI on a digitized slice (data plane) was coronally created with 5.0–6.0 mm of mesiodistal length with 5.0–6.0 mm buccolingual width. After creating ROIs up to 5.0 mm height, the interpolation of created ROIs could generate a volume-of-interest (VOI), which could be defined as the total volume (TV). Within TV, bone regeneration was quantitatively analyzed to derive bone parameters such as bone volume fraction (%; bone volume/TV), bone mineral density (g/cm^3^; bone mass/TV), and tissue mineral density (g/cm^3^; bone mass/bone volume) in Figure 4. White dash-lined boxes represented ROIs.

## Abbreviations

PDL	Periodontal ligament
3D	Three-dimensional
AM	Additive manufacturing
P2	The 2nd premolar tooth
P3	The 3rd premolar tooth
P4	The 4th premolar tooth
PCL	Poly-ε-caprolactone
FE-SEM	Field emission scanning electron microscope
IM	Intramuscularly
IV	Intravenous
KCl	Potassium chloride
Micro-CT	Micro-computed tomography
HU	Hounsfield Unit
VOI	Volume of interest
H&E	Hematoxylin and eosin
DCN	Decorin
HRP	Horseradish peroxidase
DAB	Diaminobenzidine
IHC	Immunohistochemistry
ANOVA	Analysis of variance
BVF	Bone volume fraction
BMD	Bone mineral density
BV	Bone volume
BC	Bone content
TV	Total volume
ROI	Region of interest
TMD	Tissue mineral density
