# Supplementary material for: Spatial Platform for Periodontal Ligament Angulation and Regeneration: In Vivo Pilot Study
Source: J Funct Biomater. 2025 Mar 13;16(3):99. doi: 10.3390/jfb16030099 (PMC11942940; doi:10.3390/jfb16030099)
Supplement: Supplementary file 1 [file jfb-16-00099-s001.zip › jfb-3508431-supplementary.pdf]

Journal of
Functional Biomaterials

Article

Spatial Platform for PDL Angulation and Regeneration: *In-Vivo* Pilot Study

Min Guk Kim [1,6], Do-Yeon Kim [2,3], Hyoung-Gon Ko [4], Jin-Seok Byun [2,5], Joong-Hyun Kim [7,*], Chan Ho Park [1,2,6,*]

[1] Department of Dental Biomaterials, School of Dentistry, Kyungpook National University, Daegu, 41940, Republic of Korea; minguk.kim@knu.ac.kr (M.G.K.); chanho@knu.ac.kr (C.H.P.)
[2] Department of Dental Science, Graduate School, Kyungpook National University, Daegu, 41940, Republic of Korea; dykim82@knu.ac.kr (D.-Y.K); jsbyun@knu.ac.kr (J.-S.B.); chanho@knu.ac.kr (C.H.P.)
[3] Department of Pharmacology, School of Dentistry, Kyungpook National University, Daegu, 41940, Republic of Korea; dykim82@knu.ac.kr
[4] Department of Oral Anatomy and Developmental Biology, College of Dentistry, Kyung Hee University, Seoul, 02447, Republic of Korea; hgko@khu.ac.kr
[5] Department of Oral Medicine, School of Dentistry, Kyungpook National University, Daegu, 41940, Republic of Korea; jsbyun@knu.ac.kr
[6] Advanced Dental Device Development Institute (A3DI), Kyungpook National University, Daegu, 41940, Republic of Korea; minguk.kim@knu.ac.kr (M.G.K.); chanho@knu.ac.kr (C.H.P.)
[7] Non-Clinical Evaluation Center, Osong Medical Innovation Foundation (KBIO Health®), Cheongju, Chungbuk, 28160, Republic of Korea; wndgus@kbiohealth.kr
* Correspondence: chanho@knu.ac.kr (C.H.P.); wndgus@kbiohealth.kr (J.H.K.); Tel.: +82-53-660-6890 (C.H.P.); +82-43-200-6890 (J.H.K.)

Academic Editor: Firstname Lastname

Received: date
Revised: date
Accepted: date
Published: date

Citation: To be added by editorial staff during production.

Copyright: © 2025 by the authors. Submitted for possible open access publication under the terms and conditions of the Creative Commons Attribution (CC BY) license (https://creativecommons.org/licenses/by/4.0/).

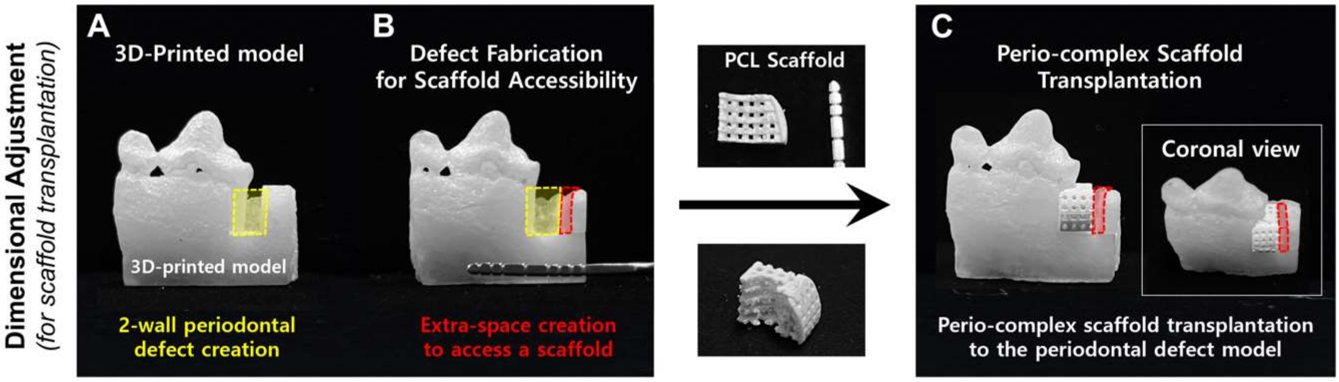

Figure S1. Optimization of defect dimension for scaffold accessibility. To avoid the destruction of perio-complex scaffolds during the transplantation, the preliminary mock surgery was executed. (a) The 3D-printed model was manufactured with the digital designed model (yellow area) and (b) extra-space was created to access a scaffold (red area). By practical testing, (c) it was determined that an extra-space of 2.0 mm (red) roughly facilitated scaffold transplantation without damaging the PDL-guiding architectures in a perio-complex scaffold.

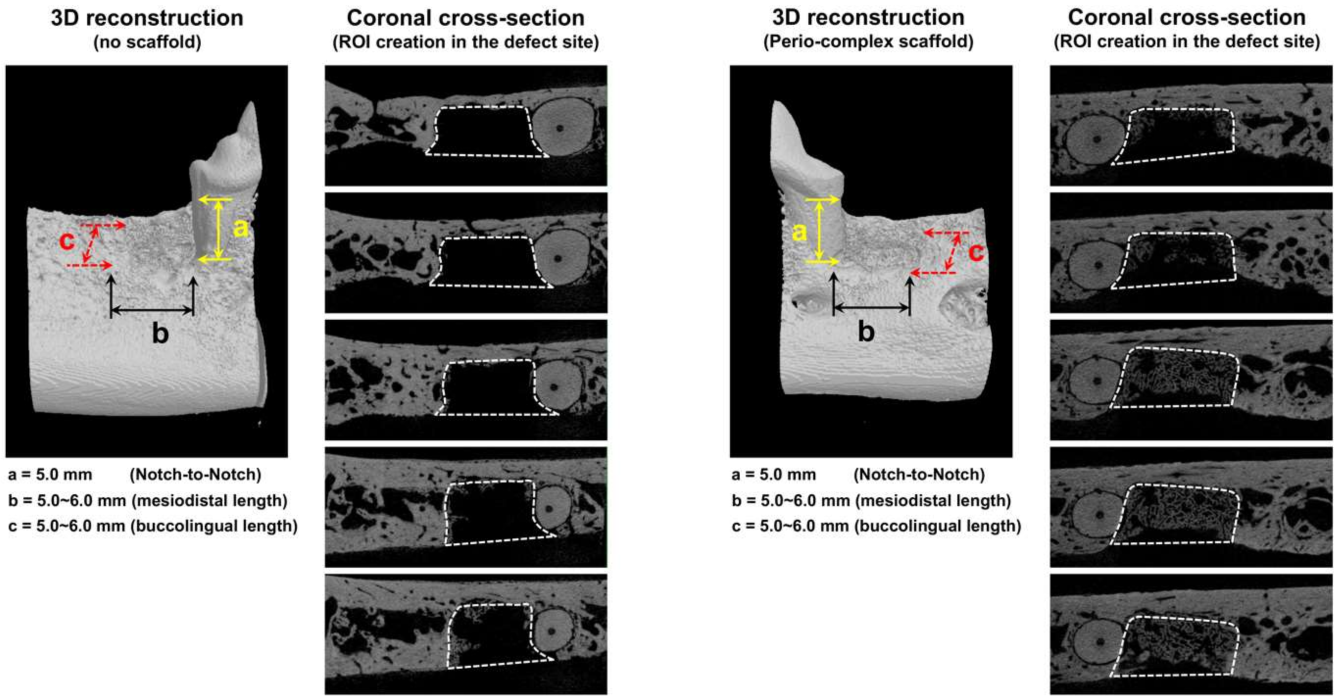

Figure S2. The creation of region-of-interest (ROI) in the 2-wall periodontal defect sites in no scaffold and perio-complex scaffold groups. From the tooth-root, ROI on a digitized slice (data plane) was coronally created with 5.0-6.0 mm of mesiodistal length with 5.0-6.0 mm buccolingual width. After creating ROIs up to 5.0 mm height, the interpolation of created ROIs could generate a volume-of-interest (VOI), which could be defined as the total volume (TV). Within TV, bone regeneration was quantitatively analyzed to derive bone parameters such as bone volume fraction (%; bone volume/TV), bone mineral density (g/cm3; bone mass/TV), and tissue mineral density (g/cm3; bone mass/bone volume) in Fig. 4. White dash-lined boxes represented ROIs.
